# Vision of objects happens faster and earlier for location than for identity

**DOI:** 10.1016/j.isci.2024.111702

**Published:** 2024-12-27

**Authors:** Christian H. Poth, Werner X. Schneider

**Affiliations:** 1 Neuro-Cognitive Psychology, Department of Psychology, Bielefeld University, Bielefeld, Germany

**Keywords:** Sensory neuroscience, Cognitive neuroscience

## Abstract

Visual perception of objects requires the integration of separate independent stimulus features, such as object identity and location. We ask whether the location and the identity of an object are processed with different efficiency for being consciously recognized and reported. Participants viewed a target letter at one out of several locations that were terminated by pattern masks at all possible locations. Participants reported the location of the target and/or its letter identity. Report performance as a function of the target duration before the mask is enabled to estimate the speed of visual processing and the minimum duration for processing to start. Visual processing was faster and started earlier for spatial location than for object identity, even though the processing of the features was (stochastically) independent. Together, these findings reveal an intrinsic preference of the human visual system for the perceptual processing of space as opposed to visual features such as categorical identity.

## Highlights

• We compared the visual processing of spatial location and identity (object category)
• Visual processing started earlier for location than for identity
• Visual processing speed was higher for location than for identity
• This suggests an intrinsic preference for the visual system for processing space

## Graphical abstract

**Figure:**
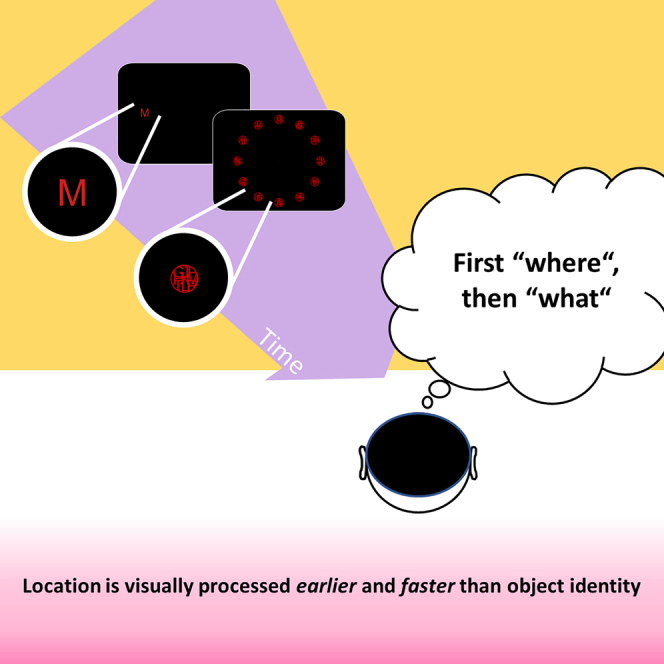

## Introduction

Human behavior is largely guided by vision. Humans visually sample the environment, they visually acquire information about objects that are relevant for current needs and behavioral goals. The visual system in the human brain encodes the different features of an object, such as form and color, in separate, specialized sub-systems.^1^ However, perceiving a coherent visual world, and guiding behavior accordingly, requires that the separate features of an object are integrated into one coherent representation.^2^^,^^3^^,^^4^^,^^5^ Perception is assumed to happen once the features become represented as object files^6^ or in visual working memory,^7^^,^^8^ a capacity-limited system for retaining (and cognitively operating on) information available even after it has disappeared from the environment.^9^ Up until this point, the different features of all objects within an eye fixation are assumed to be processed independently,^6^^,^^7^^,^^10^ in line with the distributed neural centers specializing in the processing of different features.^1^^,^^11^^,^^12^ A capacity limit in terms of object processing is nevertheless assumed by influential current theories of visual object processing.^8^^,^^13^^,^^14^ To a part, this competition is decided by attentional prioritization: processing of an object (or feature) can be enhanced based on the physical salience and/or the current task-relevance of the features of an object.^3^^,^^8^^,^^15^ Taking the top-down task-relevance aside, it is still unclear, however, whether visual perception is an intrinsic bottom-up preference for processing certain features rather than other features.

Some evidence suggests that visual features differ in a bottom-up fashion in terms of how they are processed in the visual system. In whole report paradigms with backward masking, visual features marking object boundaries (such as shape) seem to be reported more accurately than surface features as color (given equal task-relevance).^10^ In contrast, in paradigms based on feature changes, surface features as color seem to be processed for conscious perception before visual motion.^16^^,^^17^ In terms of intrinsic processing differences, the feature *location* is an especially informative case. The spatial location of an object is implicitly represented throughout the levels of the visual system in a topographic/retinoptic manner in various cortical maps,^1^^,^^18^^,^^19^ and is thought to help distinguish visual inputs from different objects,^3^ to enable sensorimotor action upon the objects,^5^ and to modulate ongoing action fast and automatically.^20^^,^^21^ In contrast to the spatial location, surface features, such as color, form or shape, and object category are represented by more specialized neural channels, centers, and maps.^1^^,^^18^^,^^22^^,^^23^^,^^24^ Thus, even though these features are ultimately bound to achieve a coherent object representation,^3^ for themselves they do not receive such an omnipresent representation as space. In line with a prominent position of spatial processing, it is well-established that spatial processing can have strong modulating effects on vision in general, namely by guiding attention to prioritize the processing of visual information from specific locations in the visual field.^25^^,^^26^^,^^27^^,^^28^ Besides this functionally important role of implicit spatial processing for initial visual processing and for guiding attention,^3^ it is unknown whether space itself also receives priority over other visual features for explicit visual recognition and report. In particular, since attention is often studied in visual search tasks requiring speeded manual actions,^29^ a seemingly high priority of spatial processing could arise from a privileged access of space to action control based on the “fast” dorsal visual system after which the “slower” ventral visual system mediating conscious perception lags behind.^20^^,^^30^^,^^31^ Thus, even though the special status of spatial processing is recognized,^27^^,^^28^^,^^32^ current theories often remain neutral regarding the intrinsic efficiency with which different classes of visual features are processed^8^^,^^33^ or might even question it.^34^

**Figure 1:**
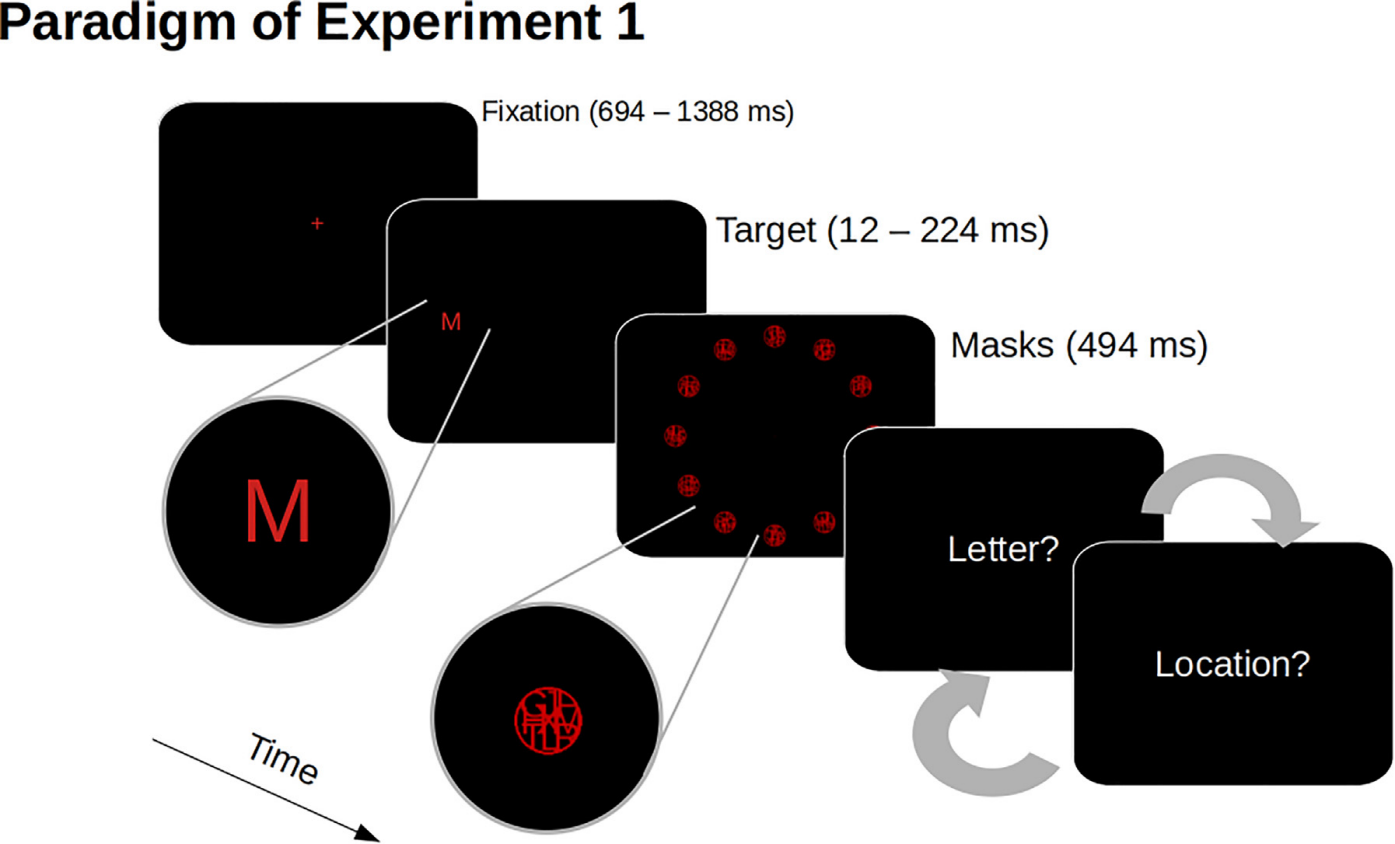
Paradigm of Experiment 1 After fixating a fixation cross, a single letter target was shown briefly at one out of 12 locations and was followed by pattern masks appearing at all 12 possible locations. At the end of a trial, participants reported the letter identity and the location (the order of these two report types was randomized and counterbalanced across trials).

Here, we ask whether the spatial location and the identity (object category) of an object are processed with different efficiency for being consciously recognized and reported. To this end, we showed observers single target letters for brief durations (terminated by pattern masks) and at different locations (Figure 1). In Experiment 1, observers reported the location as well as the letter identity of the target on a given trial. In Experiment 2, observers performed different blocks of trials in different sessions, in which they only reported the location or the identity of the target. Based on Bundesen’s^7^ Theory of Visual Attention (TVA), we modeled observers’ report performance as a psychometric function of the presentation duration of the target and estimated two key parameters of visual processing, namely the temporal threshold of visual perception, which is the presentation duration needed for visual processing to start, and the speed of visual processing in terms of objects per seconds. According to Bundesen,^7^ these two parameters determine conscious perception.

If conscious visual perception was generally better for spatial location than for object identity, performance in reporting location should be higher than for reporting object identity, across the different target durations and despite the same level of task relevance. Moreover, if location and object identity were processed independently, then participants’ reports of these two object features should be stochastically independent.^10^ In terms of the two TVA parameters of visual processing, we tested if the spatial location of an object was processed for perception with higher efficiency than the identity of the object. If so, then the visual processing speed should be higher for the location than for the object identity. In addition, if the processing of the spatial location of the object started earlier than the processing of the object identity, then the temporal perception threshold should be lower for the location than for the object identity.

## Results

The data were analyzed using custom scripts written in R (4.3.1.).^35^ The data and analysis code can be found online at (Open Science Framework: https://osf.io/jpcu4/) and contains all used R-packages. Statistical comparisons were conducted using repeated-measures analyses of variance, paired (or one-sample) *t-*tests (with Cohen’s d_z_ as effect size), followed up upon by Bayesian *t*-tests (with a prior scale of r = 0.707) yielding the Bayes Factor in favor of the alternative hypothesis (BF_10_).^36^

### Experiment 1

**Figure 2:**
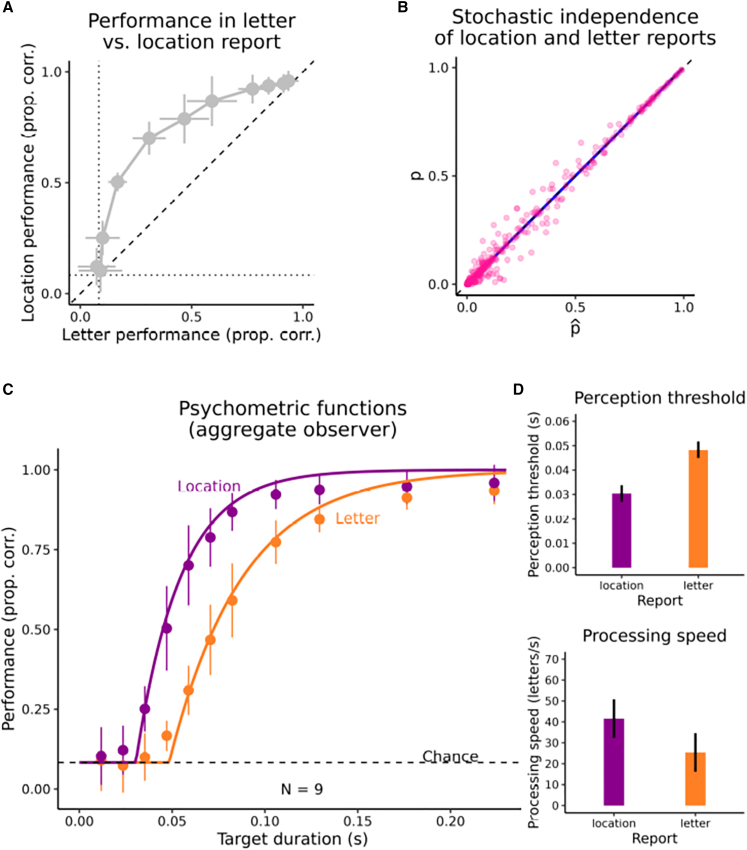
Results of Experiment 1 (A) Location report performance vs. letter report performance. Points indicate observers’ mean proportion correct, error bars the corresponding 95% confidence intervals for within designs.^37^ (B) Observed probabilities of reporting location or letter identity correctly or wrong as a function of the probabilities predicted by the observed marginal probabilities under the assumption of stochastic independence (each point represents one such probability pair for one observer and target duration). The diagonal (dashed) indicates the identity of predicted and observed probabilities (hence stochastic independence), the regression line is shown on top of it in blue. (C) Psychometric function of the aggregate observer for location vs. letter report performance as a function of target presentation duration. Points represent mean proportion correct across observers (with error bars indicating 95% confidence intervals,^37^ smooth curves indicate the psychometric functions found by averaging the parameters of the individual observers’ fitted psychometric functions. (D) Means of observers’ temporal perception thresholds and visual processing speed for perceiving location and letter identity, respectively. Error bars provide 95% confidence intervals.^37^

The stochastic independence of location and letter reports was assessed as follows (see Figure 2A for the letter and location report performance).^10^ We computed the predicted probabilities of reporting location or letter identity correctly or wrong assuming they were mutually independent, based on the observed marginal probabilities for each target duration and each observer.^10^ Across observers, there was a high correlation between these predicted probabilities and the probabilities that had been observed (Figure 2B). The mean correlation was 0.99 (*SD* = 0.014), and significantly larger than 0, *t*(8) = 207.63, *p* <0.001, *d*_z_ = 69.21, BF_10_ > 2.91∗10^12^. This shows that location reports and letter identity reports are stochastically independent.

Location reports were 16.7% (mean of observers’ mean performance differences for each target duration) more accurate than letter identity reports (Figure 2A), and this was significantly larger than 0, *t*(8) = 10.248, *p* < 0.001, *d*_z_ = 3.416, *BF*_01_ = 2685.519. To investigate these performance differences more closely in terms of temporal perception threshold and visual processing speed, the individual observer’s report performance for the two report conditions was assessed as a function of target duration, and this psychometric function was modeled as an exponential approach of perfect performance^7^ (Figure 2C shows the psychometric function for the aggregate observer):

$$p\left(t\right)=chance,if\;t<t_{0}$$

$$\;p\left(t\right)=\left\{\begin{array}{l} 1-exp\left(-v*\left(t-t_{0}\right)\right)+exp\left(-v*\left(t-t_{0}\right)\right)*chance,\;if\;t\geq t_{0}\\ chance,\;if\;t<t_{0} \end{array}\right)\text{,}$$

where p is the probability of correct report, *t*_0_ is the temporal threshold of perception, *v* is the processing speed, and *chance* is the probability of guessing correctly (here 1/12). Psychometric functions were fit using custom code (inspired by quickpsy,^38^^, cf.^^39^).

In these psychometric functions, the TVA parameter *t*_0_ is the temporal threshold of perception, that is, the target duration (in s) necessary for increasing performance over chance (i.e., the target duration where the curves in Figure 2C rise from chance) and which represents the time needed for visual processing to start.^7^ The TVA parameter v is the visual processing speed in the number of objects that can be processed per second,^7^ that is the exponential rate (i.e., the steepness) of the curves in Figure 2C).

Across observers, the temporal threshold of perception was significantly lower for reporting the location of a target compared with its letter identity (Figure 2D), *t*(8) = −8.667, *p* < 0.001, *d*_z_ = −2.889, *BF*_10_ = 929.503. Thus, the processing of location started earlier than the processing of letter identity. Likewise, visual processing speed was significantly higher for location than for letter identity (Figure 2D), *t*(8) = 2.868, *p* = 0.021, *d*_z_ = 0.956, *BF*_10_ = 3.561. Thus, the visual processing of the location not only started earlier but also proceeded faster than the processing of the letter identity.

For Experiment 1, 2 (location vs. letter identity report) x 2(report order, location vs. letter first) repeated-measures analyses of variance (ANOVA) neither showed main effects nor interaction (with report type) effects of report order on the temporal perception thresholds, *F*s(1, 8) < 3.453, *p*s > 0.100, η_G_^2^s < 0.021. However, the ANOVA showed a main effect of report order on visual processing speed, *F*(1, 8) = 6.447, *p* = 0.035, η_G_^2^ = 0.028 (Figure S1, and again, no interaction, *F*(1, 8) = 2.313, *p* = 0.167, η_G_^2^ = 0.013). Holm-corrected post-hoc tests indicated this was due to a higher visual processing speed for location than for letter identity when location had to be reported first, *p* = 0.042. Likewise, it was due to a higher visual processing speed for location when location had to be reported first as compared with the processing speed for letter identity when the letter identity had to be reported first, *p* = 0.027. This finding might suggest that the location information in working memory might decay over time depending on target duration (e.g., intermediate target durations could suffice for encoding into short-term memory but resulted in representations there still vulnerable to decay) and were fully available only when it was used for report first, without intervening letter identity report.

Conversely, for the letter identity report, and intervening location report did not seem to have any effects (Figure S1). In line with such an effect of report order, one might argue that observers strategically prioritized location over letter identity for visual processing and retention in short-term memory throughout the experiment, since both features were to be reported on every trial. Therefore, Experiment 2 asked observers to report only one of the two features in a given experimental block, so that observers could fully prioritize the target feature on a given trial. This manipulation should create conditions of equally high relevance for location and identity, ruling out top-down preferences for one feature (location) over the other (identity).

### Experiment 2

In line with Experiment 1, observers’ location reports were 16.6% (mean of observers’ mean performance differences for each target duration) more accurate than their letter identity reports, *t*(8) = 7.281, *p* < 0.001, *d*_z_ = 2.427, *BF*_10_ = 321.221 (Figure S2).

**Figure 3:**
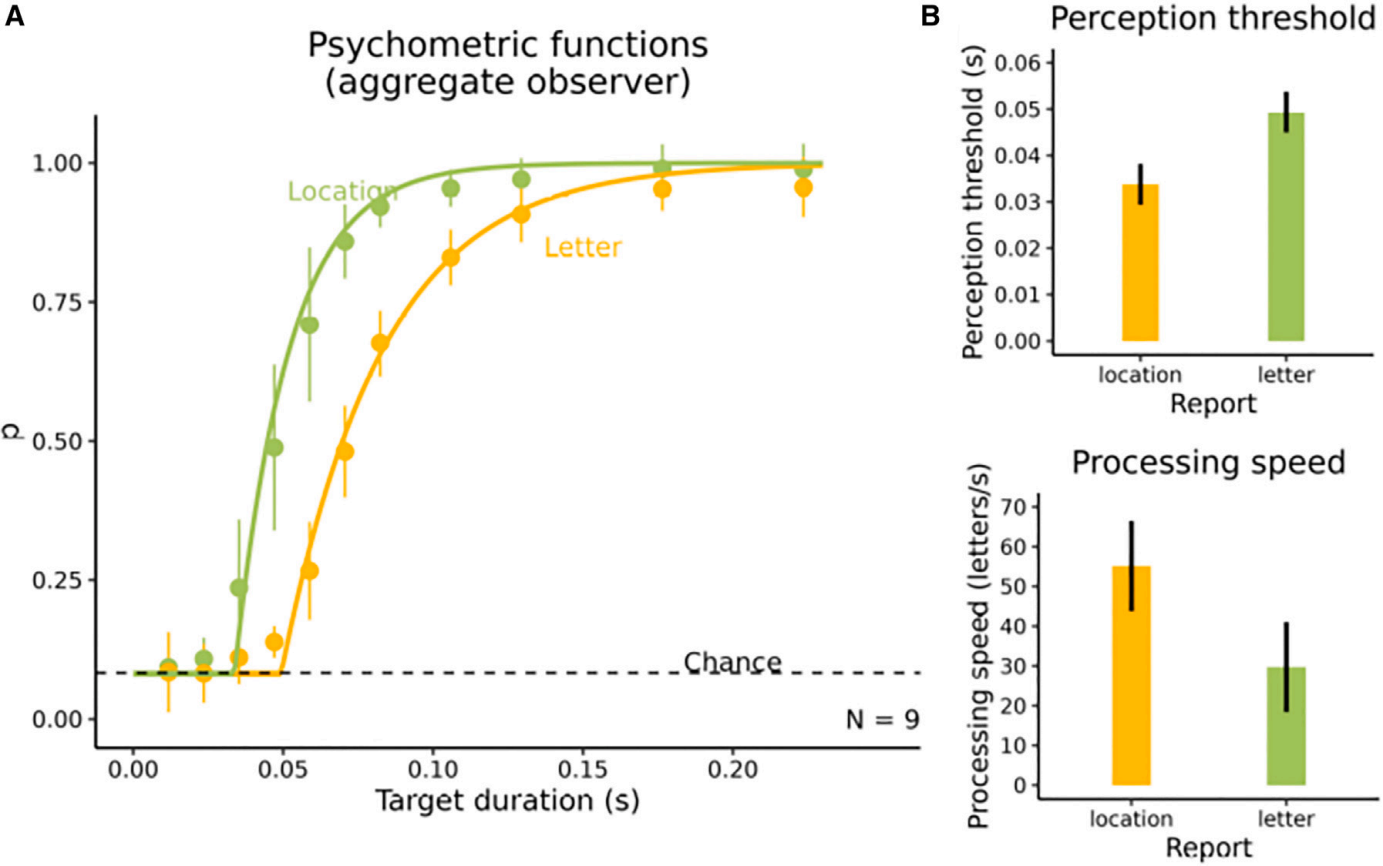
Results of Experiment 2 (A) Psychometric function of the aggregate observer for location vs. letter report performance as a function of target presentation duration. Points represent the mean proportion correct across observers (with error bars indicating 95% confidence intervals^37^), and smooth curves indicate the psychometric functions found by averaging the parameters of the individual observers’ fitted psychometric functions. (B) Means of observers’ temporal perception thresholds and visual processing speed for perceiving location and letter identity, respectively. Error bars provide 95% confidence intervals.^37^

Figure 3A shows the psychometric functions for location and letter identity reports for the aggregate observer. As in Experiment 1, observers’ temporal perception thresholds were significantly lower for location reports than for letter identity reports (Figure 3B), *t*(8) = −5.7879, *p* < 0.001, *d*_z_ = −1.929, *BF*_10_ = 86.019. Again, visual processing for location perception started earlier than processing for letter identity perception. Also, the visual processing speed for location perception was significantly higher than for the perception of letter identity (Figure 3B), *t*(8) = 3.661, *p* = 0.006, *d*_z_ = 1.220, *BF*_10_ = 9.089. Thus, when location and letter identity reports were blocked, location was still processed earlier and faster than letter identity.

## Discussion

We asked whether the bottom-up processing of the spatial location and the identity (object category) of an object were processed with different efficiency for being consciously recognized and reported. Both of our experiments demonstrate that this is the case. Overall, perceptual performance was higher for the spatial location than for the object identity. Observers’ reports of the two features were stochastically independent of one another, in line with previous findings and the assumption that visual features are processed independently and in parallel in general.^7^^,^^10^ Most importantly, we found that the bottom-up processing of the two features for visual perception/for report was differently efficient. Visual processing speed was higher for the spatial object location than for the object identity. Likewise, the temporal perception threshold was lower for location than object identity. Thus, the processing of location not only proceeded faster, but it also started earlier as compared with the processing of object identity.

One could ask if the differences between location perception and object identity perception reflected peculiarities of the task, namely, that is was merely more difficult to discriminate the letter identities as compared with the letter locations. Arguing against this idea, both, the location report and the identity report approached an asymptote near perfect performance at the highest presentation durations, showing that for both report features, there was little confusability (Figures 2C and and 3A).

In contrast to reaction time measures from speeded tasks that conflate perceptual, response, and motor processing,^40^^,^^41^^,^^42^, our paradigm offered unlimited time for responding to allow response and motor processing to finish always. Floor or ceiling effects on performance were prevented by terminating the visual presentation duration using backwards pattern masks, which are assumed to interrupt processing and extinguish visual sensory (iconic) memory.^43^ Visual processing speed and the temporal perception threshold were assessed by studying how report performance improved with the increasing presentation duration of the target. As is often done in TVA-based paradigms,^44^^,^^45^^,^^46^^,^^47^ participants viewed a single target that was terminated by a pattern mask. Crucially, the single letter was accompanied by several pattern masks at all possible target locations. Here, this was done because presenting a single mask would have directly delivered information about the target location even at the lowest target durations (which would have precluded the estimation of visual processing speed and the temporal perception threshold for spatial location). One might argue that perception in such a paradigm with post-masked targets might not only depend on the target and its presentation duration but also on the characteristics of the mask that decide how well the features of the target can be temporally segregated from the mask.^22^^,^^48^^,^^49^^,^^50^^,^^51^ The temporal segregation is assumed to rely on candidate object representations (proto-objects) that can be filtered by means of (object-based spatial) attention.^50^ The computation of attentional priorities for candidate objects as well as their initial figure-ground segregation is assumed to happen in a first, unselective (“pre-attentive”) processing phase that should contribute to the time needed to start the visual processing of objects and object features, that is, to the temporal perception threshold.^8^^,^^40^^,^^51^^,^^52^^,^^53^ Thus, our findings of lower temporal perception thresholds for spatial location than for object identity could suggest that the feature-specific masking strength (feature-specific similarity of mask and target) was higher for identity (i.e., alphanumeric category) than for location. However, such a masking-based view on the temporal threshold cannot explain why visual processing speed was also higher for location than for object identity, because visual processing is assumed to take place after the temporal segregation of target and mask and the computation of candidate target objects, and thus after the temporal perception threshold had been passed.

In Bundesen’s^7^ Theory of Visual Attention, the higher visual processing speed for location than identity could be due to two factors. First, the sensory evidence for location could be higher than for identity for at least two reasons. Large parts of the visual system are organized spatiotopic or retinotopic^18^^,^^19^^,^^cf.^ ^13^, so that space is implicitly encoded ubiquitously in the visual brain so that even subtle spatial input can successfully be matched against these vast representations in the recognition process. Spatial location (and retinotopy in general) is assumed to serve as an implicit organizing feature for guiding attention and for combining different visual features to coherent object representations^3^^,^^5^^,^^15^ and for controlling sensorimotor action,^20^^,^^21^^,^^54^ the present findings are the first to indicate a special status of spatial location also for conscious perception and explicit report. Second, the visual brain could have an intrinsic and fixed bias for categorizing objects as being at a certain location in the visual field as opposed to categorizing them as having any other feature such as a certain identity. The representation of object positions in space is often assumed to be the basis of attentional allocation,^15^^,^^55^ so that the preceding space computation prior to visual feature computations is not unlikely.^56^ So, both considerations suggest that visual processing speed for location would be higher than for other features. Rather than arising accidentally, one may speculate that prioritizing location in action control^cf.^^20^^,^^21^ and visual consciousness was itself functional, grounding representations for both processes in a common computational space integrating online sensorimotor action control and conscious perception for report. This enabled interactions between the two processes, which could be mediated by common “early” attentional processes^5^ or by two interacting visual processing streams.^57^

Akin to a higher visual processing speed, we also found that visual processing started earlier for location than for object identity, as evident from a lower temporal perception threshold. This finding is surprising because the temporal perception threshold is assumed to be unspecific to the visual features and to apply likewise to all visual features and objects.^7^^,^^58^ That is, it is assumed that the temporal perception threshold reflects “pre-attentional” processes that dissect the visual scene into preliminary representations of objects with their features,^40^ on whose basis attentional priorities (object-based attentional weights) are computed that control subsequent processing for conscious visual perception.^53^ The present findings cast doubt on this assumption of a feature-unspecific “pre-attentional” temporal perception threshold. Instead, they suggest that the start of visual processing for encoding into visual working memory and object recognition is feature-specific (or at least earlier for location).

In Experiment 1, participants reported both the location and the identity of the target after it had been presented. Thus, they had to adopt a task set in which both of the two response features were important for the task and thus received equal priority. One might argue that in such a situation, humans could have a top-down set tendency to prioritize space over identity, which would induce the above-described perceptual bias for space at the expense of identity.^7^ However, in Experiment 2, participants reported the different features in separate blocks of trials, so that here they could adopt a task-set in which the respective response feature, location or identity, was the only one of importance and thus fully prioritized. Even under these conditions, the visual processing speed and the temporal perception threshold were improved for location compared with identity. Thus, the differences between location and identity should not result from different top-down perceptual biases for response features. As such, these findings argue that the differences between location and identity were more profound, and could reflect more basic bottom-up characteristics of the visual system, such as a higher sensory evidence for location due to a stronger and more widely distributed the representation of space in the brain. This dovetails findings that location is processed faster than the surface feature of color for modifying ongoing and speeded sensorimotor actions, which may hint at a privileged access of spatial processing to mechanisms for (speeded) action control.^20^^,^^21^ In urgent situations, the most salient visual information can overpower current intentions, so that the one corresponding to the salient information out of two prepared motor plans is executed.^54^^,^^59^^,^^60^ In light of this finding, the present results might thus point to a higher intrinsic salience of location as opposed to other object features.

In sum, the present findings reveal that the spatial location of objects is preferred in visual processing for visual perception. Compared with object identity, the processing of the spatial location is more efficient, so that it starts earlier and proceeds faster. Taken together, this argues that at least for location and identity, visual processing is intrinsically different for different visual features.

### Limitations of the study

Performance in visual report tasks always bears some specificity with respect to the stimuli used. Therefore, the speed of visual processing per se cannot be assessed, only the speed for processing a certain stimulus. We used letter stimuli with specific highly effective post-masks^7^^,^^58^^,^^61^ and asked observers to report the location and/or identity of the letter, and vice versa. For our sample, we can assume that reading letters was a highly overlearned skill, so that letter identities formed distinct categories that were easy to distinguish. However, it therefore remains a question for future research, whether our differences in visual processing speed and the temporal perception threshold for identity and location were affected, if one used visual stimuli that were less overlearned and more difficult to verbalize, and thus did not belong to such distinct categories.

## Resource availability

### Lead contact

Correspondence and requests for resources should be directed to and will be fulfilled by the Lead Contact, Christian H. Poth (c.poth@uni-bielefeld.de).

### Materials availability

The computer code for running the experiments can be found here: Open Science Framework: https://osf.io/jpcu4/

### Data and code availability

• The experimental data can be found here: Open Science framework: https://osf.io/jpcu4/
• The computer code for analysis of the data can be found here: Open Science Framework: https://osf.io/jpcu4/

## Acknowledgments

We thank Josefine Albert for help with the laboratory administration. This study was supported as part of the regular research of the Neuro-Cognitive Psychology Group at Bielefeld University.

## Author contributions

Conceptualization, CHP and WXS, methodology, CHP and WXS, software, CHP, formal analysis, CHP, visualization, CHP, investigation, CHP, resources, CHP, data curation, CHP, writing – original draft, CHP, writing – review and editing, CHP and WXS, supervision, WXS.

## Declaration of interests

The authors declare no conflicts of interest.

## STAR★Methods

### Key resources table

**Table:** 

REAGENT or RESOURCE	SOURCE	IDENTIFIER
**Deposited data**		

Behavioral data	Authors	https://osf.io/jpcu4/

**Software and algorithms**		

Custom analysis scripts	Authors	https://osf.io/jpcu4/

### Experimental model and study participant details

N = 9 human observers (22 - 42 years old, MD = 25 years, 7 identifying as female, 2 as male) participated in Experiment 1 and N = 9 human observers (between 20 and 30 years old, MD = 23 years, 8 identifying as female, 1 as male) in Experiment 2. All observers had normal or corrected-to-normal visual acuity and color vision. The experiments employed within-subjects designs, so that experimental effects were assessed within observers (there were no experimental groups, which controls for between-subjects effects due to sex or gender). They were paid for participating and gave written informed consent beforehand. The experiments followed the ethical guidelines of the German Psychological Association (Deutsche Gesellschaft für Psychologie, DGPs) and were approved by the ethics committee at Bielefeld University.

### Method details

#### Apparatus and stimuli

Observers performed the experiment in a dimly lit room, with their heads fixed by a chin and head rest in a viewing distance of 71 cm to the computer monitor (ViewSonic, resolution of 1024x768 px at physical dimensions of 36x27 cm), that was pre-heated as specified previously^62^. Their eyes were tracked monocularly at 1000 Hz using a video-based and desktop-mounted eye tracker (Eyelink 1000, SR Research, Ottawa, Ontario, Canada). The experiments were programmed in MATLAB (R2014b, The Mathworks, Natick, MA, USA) using the Psychophysics Toolbox^63^^,^^64^^,^^65^ and Eyelink Toolbox^66^ extensions. Responses were collected using a QWERTZ-keyboard and a computer mouse.

Stimuli were presented against a black background (<1 cd/m^2^, measured using a Minolta LS-110, Konica Minolta, Osaka, Japan). The fixation cross was a central red “+” (RGB: [100, 0, 0]; ∼3 cd/m^2^, 0.25x0.25° of visual angle. Target stimuli were red letters (RGB: [100, 0, 0]) from the set [ABFGHJLMRSTX] (0.76x0.78°, ∼3 cd/m^2^), and the mask stimuli (100 masks per session, algorithmically created) were red circular patches of overlayed letters (see Figure 1, 0.98x0.98°, RGB: [200, 0, 0], ∼13 cd/m^2^). Stimuli were shown at one of twelve possible locations 9° around screen center. Response displays showed the text “Buchstabe?” (“letter”, 4.35x0.62°) or “Ort?” (“location”, 1.57x0.62°) in gray (∼7 cd/m^2^).

#### Procedure

Figure 1 of the main text illustrates the procedure of a single experimental trial in Experiment 1. In the beginning of a trial, observers fixated the fixation cross for a uniformly random interval between 694 and 1388 ms (in steps of 12 ms). Then, a single target letter (randomly drawn from the set of 12 letters) shown at one of the twelve possible locations (randomly drawn from the set of locations) for 12, 24, 35, 47, 59, 71, 82, 106, 129, 176, or 224 ms. The target was terminated by twelve pattern masks, one appearing at each of the twelve locations for 494 ms. Next, the response displays were presented, asking observers to report the target letter that they had seen using the keyboard or to report its location by clicking on it using the computer mouse. In Experiment 1, observers always reported both, the identity and the location of the target letter, whereby the order of the two report types was randomized and counterbalanced across trials.

In Experiment 2, the time-course of an experimental trial was the same as in Experiment 1, except that here, observers only performed one of the two report types on a trial. To this end, participants were asked either to report the location of the target letter or its identity in a block of trials.

#### Design

In Experiment 1, observers performed 11 (target durations) x 2 (location vs. letter reported first) x 25 trials = 550 trials per session. In the beginning of each session, they performed 20 practice trials. Six observers performed 4 sessions and thus 2200 trials in total. Two observers performed 2 sessions and 1100 trials each, and one observer terminated during session 3, after 1650 trials.

In Experiment 2, observers performed 11 (target durations) x 50 trials = 550 trials per session. Observers performed 4 sessions, in each of which they either reported target location or letter identity (whereby this was ordered in an ABBA or BAAB fashion, to cancel out fatigue effects of the blocks, and counterbalanced across observers). Observers performed 2200 trials in total, except for one observer who performed 1925 trials (due to a programming error).

The data, experiment code, and analysis code can be found online at Open Science Framework: https://osf.io/jpcu4/.

### Quantification and statistical analysis

The Data was analyzed using custom scripts written in R (4.3.1., R Core Team, 2023). The data and analysis code can be found online at (Open Science Framework: https://osf.io/jpcu4/) and contains all used R-packages. Statistical comparisons were conducted using repeated-measures analyses of variance, paired (or one-sample) t-tests (with Cohen’s *d_z_* as effect size and a significance criterion of α = .05), followed-up upon by Bayesian *t*-tests (with a prior scale of *r* = 0.707) yielding the Bayes Factor in favor of the alternative hypothesis (BF10).^36^ Sample size for the first experiment was estimated based on previous research,^45^^,^^61^ and the used for the second experiment that provided a replication of the first one to safeguard the reported findings against a type-I error. Within the figures, bars visualize means, and error-bars visualize 95%-confidence intervals.^37^
