# Vision of objects happens faster and earlier for location than for identity

**DOI:** 10.1016/j.isci.2025.112084

**Published:** 2025-02-22

**Authors:** Christian H. Poth, Werner X. Schneider

## Main text

(iScience *28*, 111702, February 21, 2025)

In the originally published version of this article, a typesetting error resulted in the equation "p(t) = chance, if t < t0" being included twice. This error has now been corrected in both the online and PDF versions of the article. The change does not affect the scientific conclusions of the article, and all authors agree with the correction. The publisher apologizes for any inconvenience this may have caused.

