# Supplementary material for: Vision of objects happens faster and earlier for location than for identity
Source: iScience. 2024 Dec 27;28(2):111702. doi: 10.1016/j.isci.2024.111702 (PMC11787497; doi:10.1016/j.isci.2024.111702)
Supplement: Document S1. Figures S1 and S2 [file mmc1.pdf]

iScience, Volume *28*

Supplemental information

Vision of objects happens faster

and earlier for location than for identity

Christian H. Poth and Werner X. Schneider

Supplemental materials

Experiment 1

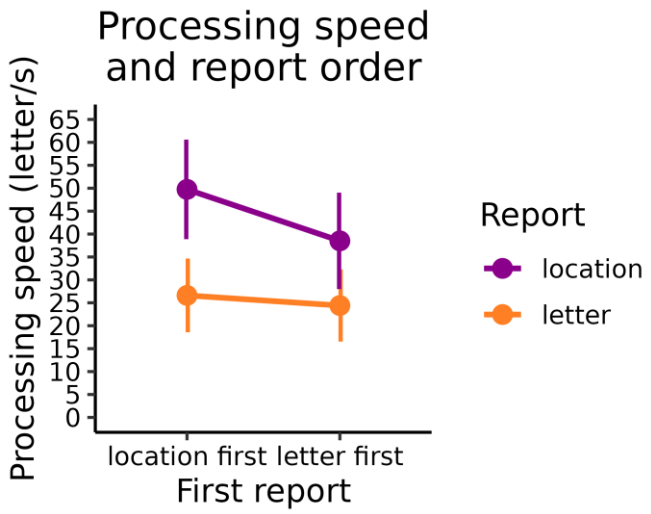

Figure S1. Observers' mean visual processing speed for location and letter reports, depending on which of the two target features was to be reported first (location first vs. letter first). Error-bars provide 95% confidence intervals[44].

Experiment 2

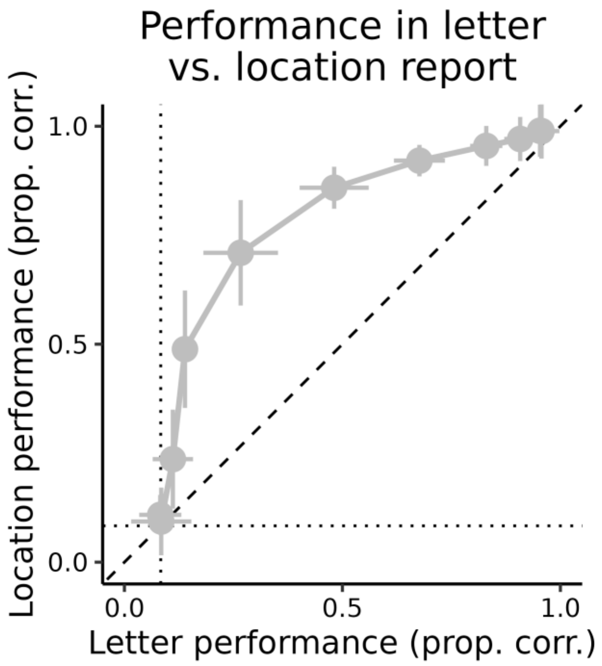

Figure S2. Location report performance vs. letter report performance. Points indicate observers' mean proportion correct, error-bars the corresponding 95% confidence intervals for within designs[44].
